# Tag-Dependent Substrate Selection of ClpX Underlies Secondary Differentiation of Chlamydia trachomatis

**DOI:** 10.1128/mbio.01858-22

**Published:** 2022-09-26

**Authors:** Nicholas A. Wood, Abigail R. Swoboda, Amanda M. Blocker, Derek J. Fisher, Scot P. Ouellette

**Affiliations:** a Department of Pathology and Microbiology, College of Medicine, University of Nebraska Medical Center, Omaha, Nebraska, USA; b School of Biological Sciences, Southern Illinois University Carbondale, Carbondale, Illinois, USA; University of Idaho; Institut Pasteur

**Keywords:** *Chlamydia*, differentiation, *trans*-translation, SsrA, tmRNA, protein turnover, protein quality control, Clp protease, ClpX

## Abstract

Despite having a highly reduced genome, Chlamydia trachomatis undergoes a complex developmental cycle in which the bacteria differentiate between the following two functionally and morphologically distinct forms: the infectious, nonreplicative elementary body (EB) and the noninfectious, replicative reticulate body (RB). The transitions between EBs and RBs are not mediated by division events that redistribute intracellular proteins. Rather, both primary (EB to RB) and secondary (RB to EB) differentiation likely require bulk protein turnover. One system for targeted protein degradation is the *trans*-translation system for ribosomal rescue, where polypeptides stalled during translation are marked with an SsrA tag encoded by a hybrid tRNA-mRNA, *tmRNA*. ClpX recognizes the SsrA tag, leading to ClpXP-mediated degradation. We hypothesize that ClpX functions in chlamydial differentiation through targeted protein degradation. We found that mutation of a key residue (R230A) within the specific motif in ClpX associated with the recognition of SsrA-tagged substrates resulted in abrogated secondary differentiation while not reducing chlamydial replication or developmental cycle progression as measured by transcripts. Furthermore, inhibition of *trans*-translation through chemical and targeted genetic approaches also impeded chlamydial development. Knockdown of *tmRNA* and subsequent complementation with an allele mutated in the SsrA tag closely phenocopied the overexpression of ClpX_R230A_, thus suggesting that ClpX recognition of SsrA-tagged substrates plays a critical function in secondary differentiation. Taken together, these data provide mechanistic insight into the requirements for transitions between chlamydial developmental forms.

## INTRODUCTION

Translational stalling is a natural occurrence for all living organisms (see reference 1 for a comprehensive review). For example, ribosomes can pause on mRNA transcripts, and it can serve regulatory functions. However, ribosome stalling also occurs after mRNA damage, frameshift mutations, and premature transcriptional termination, of which all may result in the absence of a stop codon in the mRNA template. In such instances where no ribosome release factors are recruited, the translating ribosome is effectively trapped on the transcript. Accordingly, numerous systems for the rescue of stalled ribosomes have arisen throughout evolution, as the accumulation of partially or mistranslated products can be deleterious to the organism (1–5). One such bacterium-specific system is *trans*-translation, which requires a hybrid tRNA-mRNA (tmRNA) that, with its cognate chaperone SmpB, recognizes stalled translational complexes (6–9). By providing a short coding region, tmRNA specifies the addition of a short peptide sequence, known as the SsrA tag that usually terminates with a di-alanine motif (e.g., VAA [8, 10]). The purpose of this tag is 2-fold, as follows: (i) it provides a critical stop codon to allow for release factors to bind within the ribosome and (ii) it marks the aberrant or otherwise partially translated polypeptide for degradation. The number of these tagging events underscores the importance of the *trans*-translation system for proper bacterial growth; estimates suggest that anywhere from 0.4% to 4% of all translation events end by *trans*-translation (11, 12).

While several protease systems are involved in the turnover of SsrA-tagged peptides, the Clp protease system serves as the primary factor for degradation of such tagged proteins within the cytoplasm of most eubacteria (13). ClpX typically recognizes the SsrA tag, although the specific Clp unfoldases involved in the recognition of these tagged substrates can vary depending on a number of factors (13–15). Importantly, ClpX can also recognize specific, untagged substrates either directly or with the aid of an adaptor protein (16). The AAA+ unfoldase, ClpX, forms a hexameric ring that serves as the recognition factor to unfold substrates into the proteolytically active ClpP, which functions as a 14-subunit barrel-like complex (16). ClpX specifically recognizes the SsrA tag using pore loops located within the pore of the hexameric barrel, while the positively charged RKH motif interacts with the terminal di-alanine motif to serve as the initial SsrA recognition determinant (17–20). Altering the charge of this motif by mutating any of the RKH residues to an uncharged amino acid not only potently attenuates the recognition of the SsrA tag by lowering affinity for the carboxy terminus of the tagged substrate but also shifts the substrate preference of ClpX for tag-independent (i.e., untagged) targets (21, 22). Some bacterial species also encode a specificity factor, SspB, that stimulates ClpX degradation of SsrA-tagged substrates (23–26).

Chlamydia trachomatis is an obligate intracellular human pathogen that undergoes a remarkably complex developmental cycle in which the bacteria differentiate between two functionally and morphologically distinct forms, as follows: the elementary body (EB) and reticulate body (RB) (27). The smaller, electron-dense EB is the infectious, nonreplicative form that binds to and initiates uptake into a host cell. The EB then undergoes primary differentiation into the noninfectious, replicative RB. This RB divides through a polarized budding mechanism (28), giving rise to a population of RBs. At an unknown signal mid-developmental cycle, the nascent RBs then undergo secondary differentiation into EBs. The working model for chlamydial differentiation assumes that the temporal regulation of gene expression is sufficient to trigger changes from one morphological form to the other (29, 30). However, these differences alone cannot explain the observed proteomic differences between developmental forms (31–33). Thus, we hypothesize that proteomic turnover plays a critical role during differentiation. This idea is supported by our previous work on the chlamydial ClpXP system where we observed a significant reduction in functional EB production later in the developmental cycle when ClpX function was impaired (34). We sought to expand on these studies by analyzing the chlamydial *trans*-translation system and, as such, hypothesize that the ClpX-mediated degradation of SsrA-tagged products from *trans*-translation is essential for facilitating secondary differentiation.

While *trans*-translation functions to alleviate ribosomal stalling, various organisms have co-opted this system to serve distinct functions, including coordination of replication in *Caulobacter* (35, 36), sporulation of Bacillus subtilis (37), and pathogenesis of Salmonella (38). Many bacterial species have evolved alternative modes of ribosomal rescue (e.g., ArfA/B [4, 39, 40]). However, in some pathogenic bacterial species, including *Neisseria* and Mycobacterium, *trans*-translation is essential due to the absence of any alternative rescue factors (41–43). C. trachomatis similarly lacks any identifiable alternative ribosomal rescue system (44). Furthermore, C. trachomatis does not carry a homolog to SspB or any other identified stimulatory factors (45). However, Chlamydia does carry tmRNA and SmpB, but the *trans*-translation system has not been studied in the context of chlamydial infection. Thus, we initiated studies to expand on our previous work on the ClpXP system of C. trachomatis by examining the interplay between ClpX and *trans*-translation during chlamydial development. Using *in vitro* approaches, we validated that the ClpX RKH motif mutant (ClpX_R230A_) functionally disrupted its ability to recognize a tagged substrate. We then overexpressed this mutant ClpX isoform in C. trachomatis, which revealed a substantial defect in secondary differentiation but not replication. We next determined that *tmRNA* is transcribed during mid to late development. We utilized chemical and genetic approaches to disrupt *trans*-translation and found a severely negative impact on C. trachomatis development. Using a novel knockdown system with complementation of either wild-type *tmRNA* or an allele where the degradation sequence was replaced with a 6×His tag, we observed that ribosomal rescue alone closely phenocopies ClpX_R230A_ overexpression, where replication but not secondary differentiation may still occur. These data further suggest that the recognition and degradation of a specific substrate(s) (i.e., not SsrA-tagged) by ClpX may serve a critical function in preventing the initiation of secondary differentiation. Taken together, these data provide mechanistic insight into chlamydial differentiation.

## RESULTS

### Mutation of the RKH motif attenuates ClpX recognition of the SsrA tag.

**FIG 1:**
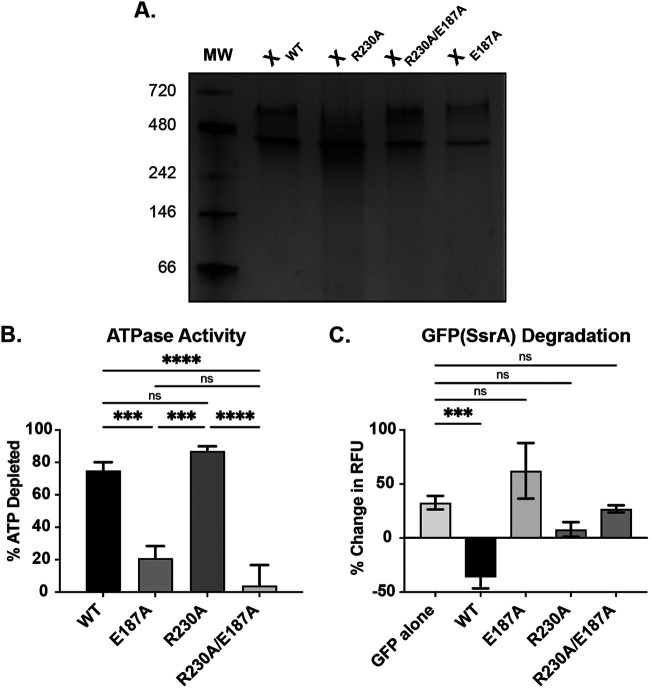
*In vitro* analysis of the ClpX R230A mutation. (A) Native-PAGE assessment of oligomerization state of the indicated ClpX isoforms. The expected size of a hexamer is ~282.6 kDa. MW, molecular weight. (B) Kinase Glo assay to assess ATPase activity of ClpX wild type (WT), Walker B mutant (E187A), RKH mutant (R230A), and double mutant (R230A/E187A). Values are reported as % of starting ATP depleted. (C) GFP(SsrA) degradation assay for the indicated ClpX isoforms. Measured as change in fluorescence from starting value. For both B and C, ***, *P* < 0.001; ****, *P* < 0.0001; and ns, not significant by ordinary one-way analysis of variance (ANOVA) with Tukey’s *post hoc* comparisons test. Data in B and C are the averages of three biological replicates. RFU, Relative Fluorescence Units.

To validate that the R230A mutation did not affect oligomerization state, we first purified recombinant chlamydial ClpX_R230A_ and utilized a native-PAGE assay (Fig. 1A). We determined that ClpX_R230A_ formed the expected hexamer as described previously for the recombinant chlamydial wild-type ClpX and Walker B ATPase mutant ClpX_E187A_ isoforms (34). Similarly, the double mutant ClpX_R230A/E187A_ was also observed as a hexamer. Next, we sought to assess the ATPase activity of ClpX_R230A_, as we and others have previously demonstrated the *in vitro* ATPase activity of wild-type and Walker B mutant isoforms of chlamydial ClpX (34, 46). Using purified recombinant ClpX_R230A_, we compared its ATPase activity to other ClpX isoforms. As expected, we measured minimal differences in total ATP hydrolysis between wild-type ClpX and ClpX_R230A_, although our assay assesses endpoint ATP levels and not necessarily the rate of hydrolysis (Fig. 1B) (47). Conversely, disrupting the Walker B motif (E187A) significantly reduced ATP hydrolysis whether alone, as previously noted (34), or in combination with the R230A mutation. We next assessed the ability of our various ClpX isoforms, in combination with the ClpP1P2 protease subunits, to recognize and degrade purified GFP tagged with a C-terminal chlamydial SsrA tag. Using wild-type ClpX, we observed a significant reduction in GFP fluorescence. However, for both the E187A and the R230A, single or double, mutant isoforms, GFP fluorescence was maintained throughout the duration of the experiment. This result is consistent with these isoforms being unable to unfold and/or recognize GFP_VAA_ (Fig. 1C). Additionally, all ClpX isoforms, including the wild type, were unable to degrade GFP_VDD_ (data not shown), which lacks the necessary residues for recognition by ClpX. These data confirm that the R230A mutation prevents chlamydial ClpX from targeting the SsrA tag without disrupting its oligomeric state or ATPase activity, supporting that ClpX should still retain adaptor activity for non-SsrA substrates.

### Overexpression of ClpX_R230A_ attenuates secondary differentiation.

**FIG 2:**
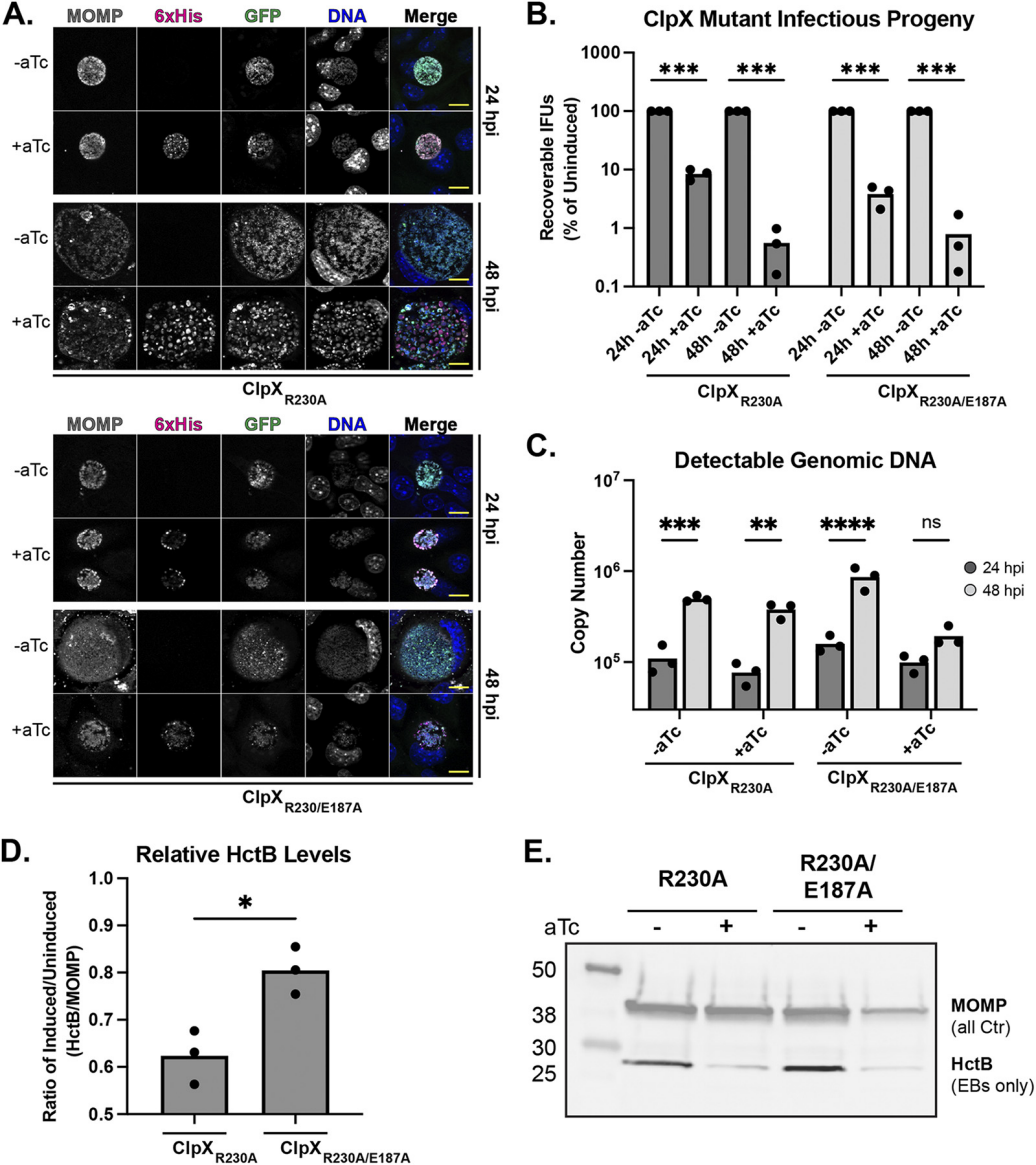
Analysis of the effects of ClpX RKH mutant isoform overexpression. (A) Indirect immunofluorescence assay (IFA) of the IFU primary infections. Scale bar, 10 μm. (B) Inclusion forming unit (IFU) analysis following overexpression of ClpX RKH mutant isoforms. Samples were induced with 20 nM aTc or not at 10 h postinfection (hpi). IFUs were normalized to uninduced values and plotted on a log scale. ***, *P* < 0.001 by multiple paired *t* tests. (C) Genomic DNA quantitation determined by qPCR of gDNA from 24 to 48 hpi. **, *P* < 0.01; ***, *P* < 0.001; ****, *P* < 0.0001; ns, not significant by ordinary two-way ANOVA with Sidak’s *post hoc* multiple-comparison test. (D) HctB levels quantitated from Western blots probing for HctB and MOMP. The ratio of HctB to MOMP was determined, and the resulting values were used for the ratio of the induced to uninduced. *, *P* < 0.05 by paired *t* test. (E) Representative Western blot used for quantitation in D. Images were selected as representative from three biological replicates. All graphs are representative of three biological replicates. aTc, anhydrotetracycline.

We demonstrated previously that the overexpression of wild-type ClpX has no significant effect on C. trachomatis, whereas overexpression of ClpX_E187A_ significantly reduced infectious progeny while having limited to no effect on replication (as assessed by genomic DNA [gDNA] levels) or accumulation of secondary differentiation markers (34). Similarly, we sought to assess the effect of overexpression of ClpX_R230A_ on C. trachomatis development compared with that of the double mutant ClpX_R230A/E187A_ using a variety of parameters. We first employed an indirect immunofluorescence assay (IFA) analysis to assess any morphological effects on inclusion size or organism numbers after overexpressing the mutant ClpX isoforms. Surprisingly, we observed that the overexpression of ClpX_R230A_ did not result in a noticeable reduction of inclusion size, and there was no apparent qualitative effect on overall organism numbers (Fig. 2A). These results are similar to what we observed previously when overexpressing the wild-type isoform (34). Conversely, we observed a reduced inclusion size following the overexpression of the double mutant, and these data were similar to prior results when the Walker B (E187A) mutant was overexpressed alone (34), suggesting the E187A mutation is epistatic to R230A. We next performed inclusion forming unit (IFU) assays to measure infectious progeny production. Contrary to the IFA data where there was no apparent qualitative effect of overexpressing the R230A isoform, we observed a substantial quantitative reduction in IFUs with overexpression of the ClpX_R230A_ single mutant at 24 h postinfection (hpi) that was exacerbated later in development at 48 hpi (Fig. 2B). This finding contrasts sharply with the effect of wild-type ClpX overexpression (34). Rather, this reduction in IFUs mirrored the overexpression of the ClpX_R230A/E187A_ double mutant (Fig. 2B) or the Walker B mutant alone (34).

To tease apart why IFU production was reduced in each of these strains, we next measured changes in genomic DNA (gDNA) and HctB production to serve as proxies for replication and EB production, respectively. The replication of the ClpX_R230A_ overexpression strain following induction was largely unaffected compared with that of the uninduced control, as noted by the increase in gDNA levels from 24 to 48 hpi. However, the double-mutant gDNA levels showed no significant difference between 24 and 48 hpi (Fig. 2C), indicating that replication is effectively stalled. When probing for the EB-specific protein HctB (48), we observed a significant reduction in HctB levels relative to major outer membrane protein (MOMP) levels following induction of ClpX_R230A_ (Fig. 2d and e). Conversely, overexpression of ClpX_R230A/E187A_ did not reduce the HctB-to-MOMP ratio equivalently, providing further evidence that the Walker B E187A mutation is epistatic to R230A and negatively impacts chlamydial development differently than the single R230A mutation. Consistent with the detectable gDNA levels, the MOMP level observed by Western blot following ClpX_R230A_ overexpression further supports that total bacterial numbers are unaffected, but the proportion of these bacteria that are EBs is reduced. Conversely, the MOMP level following ClpX_R230A/E187A_ overexpression closely reflects the drop in bacterial numbers detected by quantitative PCR (qPCR).

The normal replication coupled with the reduction in HctB levels suggests that overexpression of ClpX_R230A_ is preventing secondary differentiation. However, these data indicate two distinct possibilities as to how secondary differentiation is blocked. One possibility is altered transcriptional programming, where the transcription of genes required for EB production is not activated. The second scenario is blocked morphological transition, where transcriptional programming is unaffected but RBs are unable to differentiate into EBs on a morphological and functional level. To test these possibilities, we isolated RNA throughout the developmental cycle, starting with the time of induction of ClpX_R230A_ overexpression. We then profiled the transcription of several genes that are representative of the early, mid-, and late developmental stages of development (see Fig. S1 in the supplemental material). We observed no notable alterations of any of these transcript levels, supporting that overexpression of the R230A isoform blocks morphological differentiation. However, more comprehensive transcriptome sequencing (RNA-seq) analyses to evaluate global transcript patterns will be required to determine whether a specific gene might contribute to the observed phenotype.

10.1128/mbio.01858-22.1 FIG S1 RT-qPCR analysis of representative early (*euo*), mid- (*clpP2*), mid-late (*hctA*), and late (*omcB*, *hctB*, and *tsp*) developmental cycle gene transcripts following overexpression of ClpX_R230A_. At each indicated timepoint, RNA and genomic DNA (gDNA) were harvested and processed as described. RNA was reverse transcribed, and levels of the resulting cDNA and gDNA were determined by RT-qPCR. Values of cDNA were normalized to gDNA and plotted on a log_10_ scale. Error bars represent SEM. No differences at any timepoints were statistically significant by paired *t* test. Data plotted are the average of three biological replicates. Download FIG S1, TIF file, 0.5 MB. Copyright © 2022 Wood et al. 2022 Wood et al. https://creativecommons.org/licenses/by/4.0/ This content is distributed under the terms of the Creative Commons Attribution 4.0 International license.

### tmRNA transcription peaks mid-developmental cycle and is critical to chlamydial growth.

**FIG 3:**
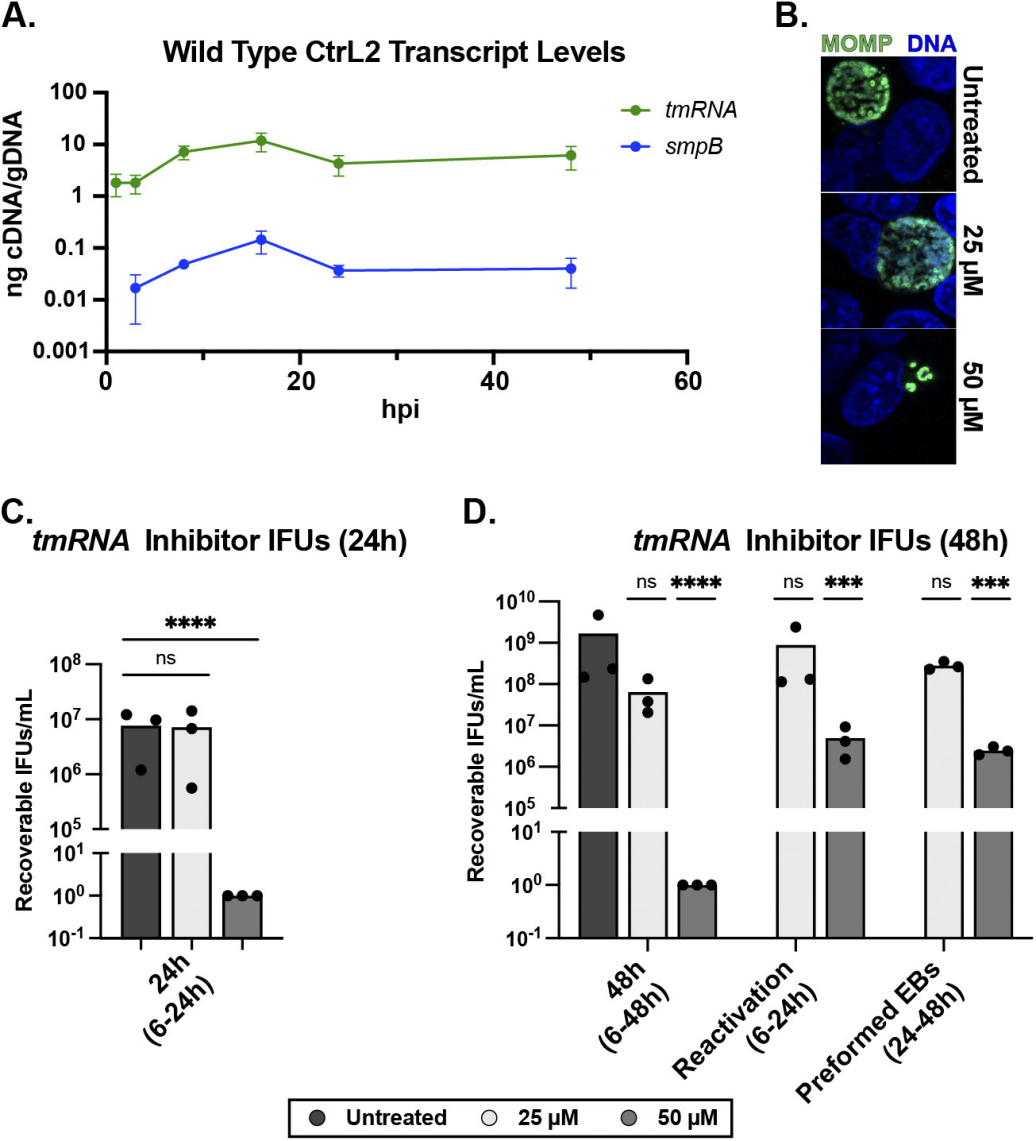
Testing the essentiality of *trans*-translation to chlamydial development. (A) Time course RT-qPCR analysis of *tmRNA* and *smpB* transcript levels. RNA and gDNA were harvested, processed, and quantitated as described previously. Values are plotted on a log_10_ scale. Data are plotted as the average of three biological replicates. (B) Immunofluorescence assay of MBX-4132 treatment at 24 hpi. Chlamydial MOMP in green, and DNA in blue. (C and D) Shown are results of 24 hpi (C) and 48 hpi (D) IFU analysis following treatment with the *trans*-translation inhibitor MBX-4132. Treatment windows are as described in the figure. Recoverable IFUs were plotted on a log_10_ scale. Values were log transformed for normalcy prior to analysis. ***, *P* < 0.001; ****, *P* < 0.0001; ns, not significant by ordinary two-way ANOVA with Dunnett’s *post hoc* multiple-comparison test; all compared with the untreated control.

Given the substantial impact of ClpX_R230A_ overexpression on C. trachomatis and the function of the RKH motif in recognizing SsrA-tagged substrates, we next shifted our focus to the *trans*-translation system. Because C. trachomatis is a developmentally regulated bacterium, we performed reverse transcriptase quantitative PCR (RT-qPCR) to assess when *tmRNA* and *smpB* are maximally transcribed. We observed transcriptional peaks at 16 hpi, supporting the conclusion that these genes are important during peak replication and translation times (Fig. 3A). We then leveraged a chemical inhibitor of *trans*-translation to assess the effects on chlamydial development. This inhibitor (MBX-4132, a KKL-35 derivative [49]), was shown previously to be highly effective in the treatment of multidrug-resistant Neisseria gonorrhoeae (50). We performed a standardized set of experiments (see reference 51) to determine the effect of this compound on chlamydial development. Continuous treatment with 50 μM potently inhibited the production of infectious progeny (Fig. 3B and C). However, transient drug treatment experiments (treating at 6 hpi, removing drug at 24 hpi, and harvesting IFUs at 48 hpi) revealed that C. trachomatis survives this inhibition and reactivates to continue development, suggesting that the inhibition of *trans*-translation is bacteriostatic to C. trachomatis (Fig. 3C). We then added the compound at 24 hpi to determine whether MBX-4132 would affect preformed EBs and found no reduction in infectious progeny. This finding suggests little to no action against EBs, which is unsurprising given our previous findings with other bacterial inhibitors (51). However, these data indicate that, overall, *trans*-translation is essential to chlamydial developmental cycle progression.

### SsrA tag recognition is critical for secondary differentiation.

**FIG 4:**
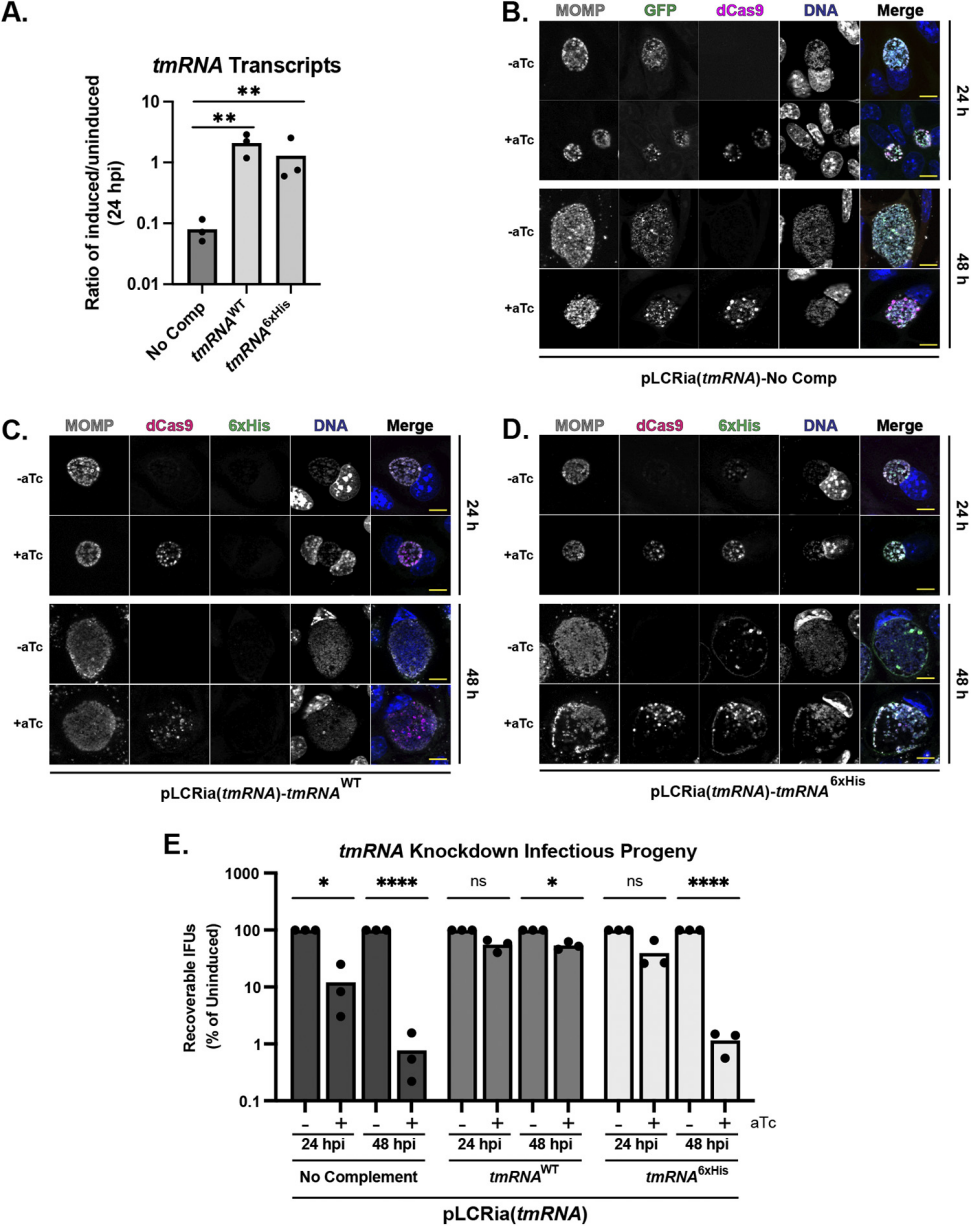
Functional *tmRNA* is critical for chlamydial development. (A) Confirmation of knockdown and complementation of *tmRNA*. Values determined by RT-qPCR were normalized to the uninduced sample and plotted. Knockdown was induced or not at 10 h postinfection (hpi). **, *P* < 0.01 by ordinary one-way ANOVA. Data are plotted from three biological replicates. (B to D) IFA analysis of no (B), wild-type *tmRNA* (C), or *tmRNA*^6xHis^ (D) complementation following knockdown of *tmRNA*. Scale bar, 10 μm. (E) Inclusion forming unit (IFU) analysis following knockdown in the indicated strains. IFUs were normalized to uninduced values and plotted on a log scale. *, *P* < 0.05; ****, *P* < 0.0001; ns, not significant by ordinary two-way ANOVA with Sidak’s *post hoc* multiple-comparison test. Data are plotted from three biological replicates.

Given that disrupting *trans*-translation resulted in no recovery of IFUs, we next utilized a CRISPR interference approach (CRISPRi) that was pioneered in the Chlamydia field by our lab to disrupt *trans*-translation on a genetic level (34, 52, 53). We designed a guide RNA (gRNA) to bind within the *tmRNA* promoter region, which resulted in >90% knockdown of detectable *tmRNA* transcripts following the induction of dCas9 expression (Fig. 4A). We next utilized a complementation approach where we encoded either a wild-type or 6×His variant of *tmRNA* (see reference 54) driven by the *incDEF* promoter on our knockdown construct (see Fig. S2 in the supplemental material) (55). We included either the wild-type *tmRNA* or a *tmRNA*^6xHis^ allele that replaces the last six amino acids of the degron with a poly-histidine tag to prevent the rapid turnover of the tagged proteins (see reference 54). This process would allow for ribosome recycling but not the degradation of the tagged peptides. We confirmed the restoration of *tmRNA* transcript levels following the induction of dCas9 expression, indicating that our strategy was successful (Fig. 4A). IFU and IFA analysis together suggested the inhibition of chlamydial developmental cycle progression after *tmRNA* knockdown (Fig. 4B and E), supporting that a reduction of *trans*-translation activity is highly detrimental to C. trachomatis. However, this strategy alone did not reveal whether the observed developmental effects resulted from unresolved ribosomal stalling or the lack of degradation of SsrA-tagged substrates. Furthermore, transcriptional programming, reflected by a subset of genes representative of the different stages of the developmental cycle, remained unaffected during *tmRNA* knockdown or after complementation with either *tmRNA* allele (see Fig. S3 in the supplemental material).

10.1128/mbio.01858-22.2 FIG S2 (A) Schematic for *tmRNA* complementation. The SsrA tag-coding region of *tmRNA* is either unchanged or altered to replace the last six amino acids with a 6xHis tag. (B and C) Plasmid arrangement for complementation with wild-type (B) or 6xHis (C) *tmRNA* variants. Created using SnapGene viewer. Download FIG S2, TIF file, 1.0 MB. Copyright © 2022 Wood et al. 2022 Wood et al. https://creativecommons.org/licenses/by/4.0/ This content is distributed under the terms of the Creative Commons Attribution 4.0 International license.

10.1128/mbio.01858-22.3 FIG S3 RT-qPCR analysis of representative early (*euo*), mid- (*clpP2*), mid-late (*hctA*), and late (*omcB*, *hctB*, and *tsp*) developmental cycle gene transcripts following induction with the indicated strain. At each indicated timepoint, RNA and genomic DNA (gDNA) were harvested and processed as described. RNA was reverse transcribed, and levels of the resulting cDNA and gDNA were determined by RT-qPCR. Values of cDNA were normalized to gDNA and plotted on a log_10_ scale. Values plotted are from three biological replicates. Download FIG S3, TIF file, 1.5 MB. Copyright © 2022 Wood et al. 2022 Wood et al. https://creativecommons.org/licenses/by/4.0/ This content is distributed under the terms of the Creative Commons Attribution 4.0 International license.

We then performed IFU and IFA experiments of the *tmRNA* complemented strains and observed that the *tmRNA*^WT^ variant complemented knockdown while *tmRNA*^6xHis^ did not (Fig. 4C to E). Moreover, the *tmRNA*^6xHis^ strain closely phenocopied the uncomplemented knockdown strain by IFUs, which supports that functional targeting (i.e., degradation) of the SsrA tag, but not necessarily ribosome recycling, is important to infectious progeny production. IFA analysis reflected that *tmRNA*^WT^ complementation restored bacterial and inclusion morphology to the wild type. Interestingly, overall inclusion size was not affected with *tmRNA*^6xHis^ complementation, although bacterial density within the inclusion appeared reduced (Fig. 4D). Interestingly, the 6×His labeling in the *tmRNA*^6xHis^ strain was detected both within the organism and around the inclusion membrane, suggesting extensive SsrA tagging of chlamydial proteins (Fig. 4D). Efforts are ongoing to identify these 6×His-tagged peptides but remain challenging due to the experimental system.

**FIG 5:**
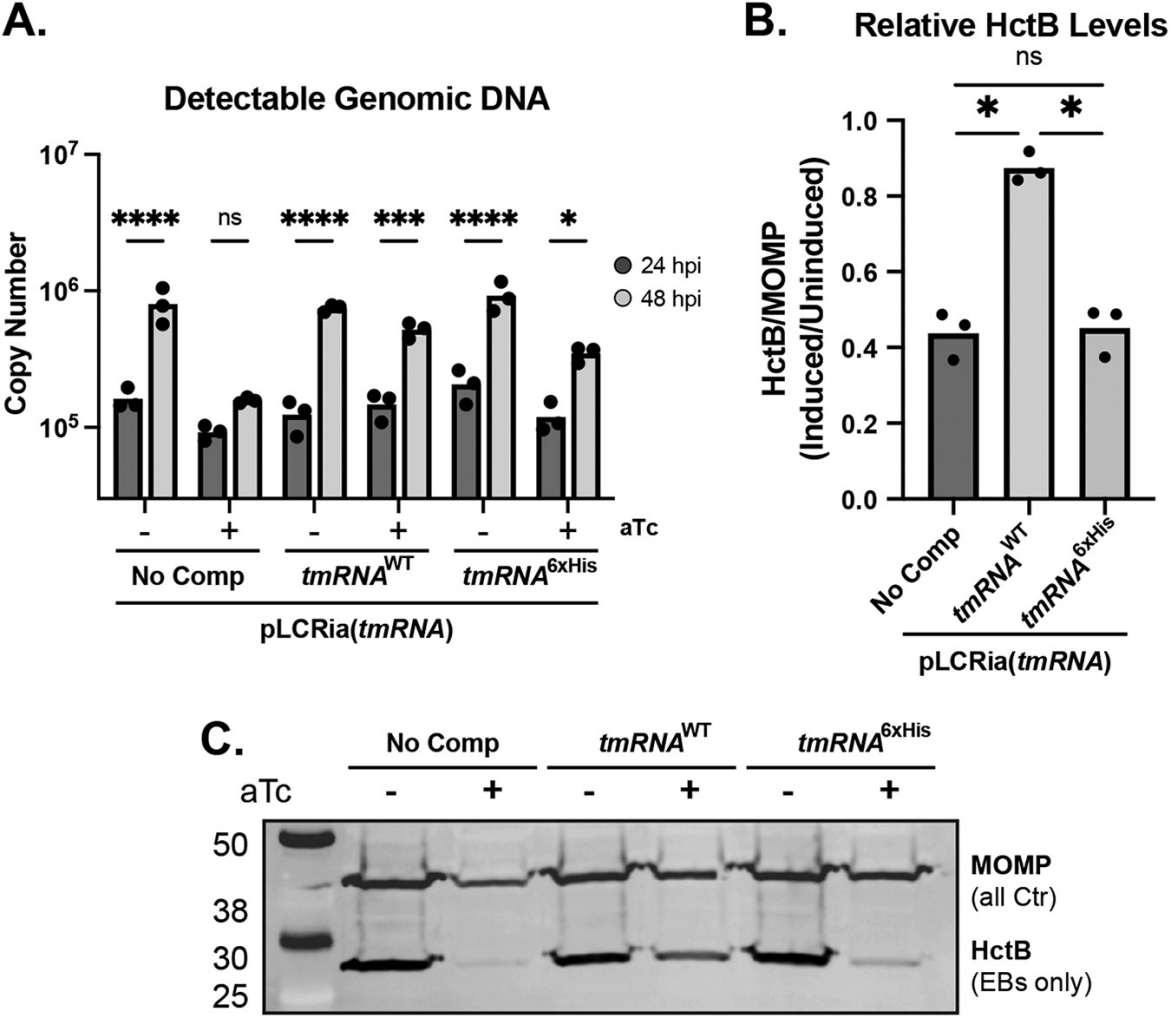
Recognition of *tmRNA* plays a role in secondary differentiation. (A) Genomic DNA quantitation determined by qPCR of gDNA from 24 to 48 hpi. *, *P* < 0.05; ***, *P* < 0.001; ****, *P* < 0.0001; ns, not significant by ordinary two-way ANOVA with Sidak’s *post hoc* multiple-comparison test. (B) HctB levels quantitated from Western blots probing for HctB and MOMP. The ratio of HctB to MOMP was determined, and the resulting values were used for the ratio of the induced to uninduced. Values are representative of three biological replicates. *, *P* < 0.05 by paired *t* test. (C) Representative Western blot used for quantitation in B. A and B are the result from three biological replicates. No Comp, no complementation.

Given that we successfully reduced *tmRNA* transcript levels using CRISPRi, we asked whether the knockdown of *tmRNA* with or without complementation would affect secondary differentiation, as we observed with ClpX_R230A_ overexpression. Knocking down *tmRNA* levels will have two principal effects, as follows: (i) a reduction of the SsrA-tagged substrate pool and (ii) widespread ribosomal arrest on nonstop transcripts. Based on published data from other experimental systems (54), we rationalized that complementation with *tmRNA*^6xHis^ would restore ribosomal rescue but not degradation of the partially translated products destined for SsrA tagging. The net effect would be to alleviate ribosome stalling while simultaneously lowering the pool of tagged substrates for ClpX to target. As such, the endogenous ClpX substrate selection would shift to untagged targets that encode degrons recognized directly by ClpX or by a putative adaptor(s). We performed a similar series of studies as described for the ClpX_R230A_ strain to assess these effects on chlamydial growth and development. These assays revealed a defect in the replication of the *tmRNA* knockdown strain following induction, supporting that the reduced capacity for *trans*-translation effectively blocks developmental cycle progression (Fig. 5A). Importantly, the replication was restored with complementation of either *tmRNA*^WT^ or *tmRNA*^6xHis^ (Fig. 5A), indicating that ribosomal rescue but not necessarily degradation of SsrA-tagged substrates is required for continued growth and division. These data further support that C. trachomatis has no alternative ribosomal rescue factors. However, these data fail to explain the observed decrease in infectious progeny with *tmRNA*^6xHis^ complementation. Probing for HctB levels uncovered a significant reduction of HctB in both *tmRNA* knockdown-only and *tmRNA*^6xHis^-complemented strains following induction (Fig. 5B and C). These lowered ratios indicate that, as a whole, fewer EBs are being produced. Importantly, the *tmRNA*^6xHis^ complementation strain closely phenocopied the sustained replication and lowered EB production of the ClpX_R230A_ overexpression strain. Taken together, these data provide evidence that *trans*-translation is essential to chlamydial survival and that the recognition of a functional SsrA tag is critical to developmental cycle progression.

## DISCUSSION

The functional and morphological differences between chlamydial EBs and RBs can, to a large degree, be explained by the proteomic variability between developmental forms. However, the mechanisms for achieving this difference in protein repertoires remain unclear, and as such, we hypothesize that protein turnover plays an integral role during chlamydial differentiation. Building on our previous work studying the chlamydial Clp protease system (34), we investigated the importance of the interplay between ClpX and *trans*-translation to both chlamydial development and differentiation. Although this system functions primarily in the indiscriminate rescue of stalled ribosomes and translational quality control, a growing body of evidence supports more diverse and specific roles in many bacteria, where *cis-*encoded transcriptional elements may facilitate stalling under specific circumstances (35–37, 41, 56). Based on our data, Chlamydia is no exception.

**FIG 6:**
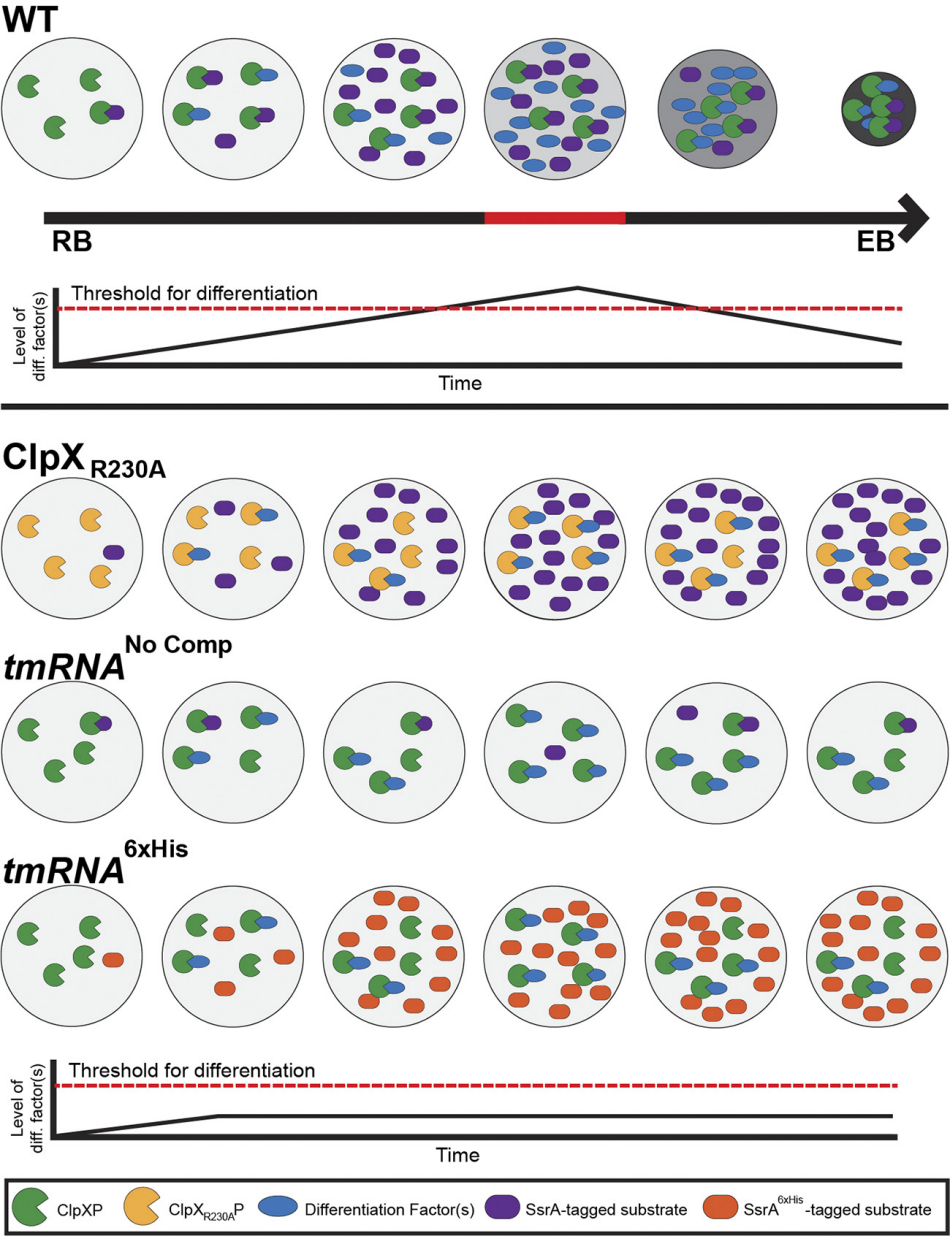
Probabilistic model of the roles of ClpX and *trans*-translation during differentiation. In this model, ClpX targets both tagged and untagged substrates. As *trans*-translation events accumulate, the level of available SsrA-tagged substrates increases, effectively reducing the number of ClpXP complexes that can target factors involved in mediating secondary differentiation. Once the level of differentiation factor(s) reaches the threshold, commitment to secondary differentiation on a morphological level occurs. The effects of the various constructs on this model are shown at the bottom, where the differentiation factor(s) level never reaches the threshold to trigger morphological secondary differentiation.

Here, the utilization of *trans*-translation by C. trachomatis appears to be unique, and we speculate that this system may have been co-opted to serve as an internal timing mechanism to trigger secondary differentiation. More specifically, we hypothesize that the accumulation of SsrA-tagged substrates increases the pool of targets available to ClpX, thus titrating ClpX away from targets encoding natural degrons or those that rely on an adaptor for delivery to ClpX. We speculate one or more of these SsrA tag-independent ClpX substrates function as a secondary differentiation factor that is prevented from triggering differentiation until a threshold of SsrA-tagged substrates is breached (Fig. 6). This effect would occur in a stochastic and probabilistic fashion that depends on the overall rates of *trans-*translation and ClpX levels that is consistent with the asynchronous manner of chlamydial RB-to-EB differentiation. In support of this model, limiting ClpX substrate selection to untagged targets, either by overexpression of the RKH mutant isoform or by replacing the endogenous SsrA tag with a nontargetable variant, is sufficient to block secondary differentiation. We reason that knocking down *tmRNA* and complementing with the *tmRNA*^6xHis^ reduces the pool of degradable SsrA-tagged proteins (by ~90% based on *tmRNA* levels after knockdown) while still allowing for the liberation of stalled ribosomes, thus freeing ClpX to primarily target untagged proteins. Complementation with *tmRNA*^6xHis^ restored *tmRNA* transcripts to wild-type levels, suggesting that approximately 90% of the SsrA-tagged pool contains a 6×His in place of the normal degron. However, we are unable, due to the technical limitations of the experimental system, to quantitatively assess the levels or identity of *trans*-translated products tagged with 6×His compared with the endogenous SsrA-tagged protein pool, which remains a caveat to this model. Nonetheless, given the frequency of *trans*-translation events observed in other bacteria, we conclude that *trans*-translation may serve as a simple yet reliable mechanism that ensures programmed differentiation of C. trachomatis using a posttranscriptional mechanism.

One alternative interpretation of the data could be that a differentiation inhibitor(s) is normally SsrA-tagged and degraded to allow for the RB-to-EB transition. Presumably, this inhibitor would be a late gene product and would be functional only if the pool of SsrA-tagged substrates did not exceed a critical threshold. When *tmRNA* is knocked down and complemented with the 6×His-tagged allele, the inhibitor is still tagged but not degraded and will exert its function to block secondary differentiation. Similarly, when expressing the ClpX_R230A_ isoform, the putative inhibitor is SsrA tagged as usual but not degraded, which will lead to its accumulation to block differentiation. One question is why overexpressing wild-type ClpX does not have an impact on chlamydial secondary differentiation or growth (34). In the first model, we speculate that the stability of ClpX (as noted elsewhere [57]) is not sufficient to allow for “overdegradation” of target substrates or, alternatively, there is a limited pool of ClpP for ClpX to bind to form a functional protease. In the second model, additional wild-type ClpX would be free to recognize and degrade a putative differentiation inhibitor. Further work is necessary to distinguish between these possibilities but identifying substrates of ClpX should be helpful. These items are a major focus of our ongoing work but remain challenging due to the obligate intracellular nature of Chlamydia.

In trying to understand ClpX target specificity, we overexpressed the ClpX_R230A_ mutant in C. trachomatis, which led to an astounding phenotype of attenuated secondary differentiation. The bacteria are locked morphologically as RBs with no apparent accumulation of EBs as assessed by either microscopy or by IFU assays (Fig. 2B). Nonetheless, representative transcripts of this strain following overexpression indicate that, transcriptionally, these bacteria are developmentally unaffected (Fig. S1), meaning late genes are transcribed. These data indicate, surprisingly, that transcriptional activation of late genes may not be sufficient on its own to drive secondary differentiation as generally assumed in the field. However, more comprehensive studies are required to assess whether an individual gene(s) or other physiologic factor (e.g., a metabolite) could contribute to this effect, and it is likely that secondary differentiation may integrate multiple signals to allow for functional EB production. Regardless, our data indicate that the reduction in recoverable infectious progeny with the concomitant reduction in HctB levels signifies that secondary differentiation is halted on a posttranscriptional level. We also observed that prolonged culture of this strain with sustained induction beyond 48 hpi still results in inclusion lysis at levels virtually indistinguishable from the uninduced control cultures yet without producing EBs (Fig. 2B; unpublished observation). That lysis still occurs despite attenuated secondary differentiation suggests that infectious progeny production is not necessary for host cell egress, which is consistent with the activation of host cell pathways to mediate cell lysis (58, 59). One caveat to these data is that the endogenous ClpX is still present during the period of overexpression, which may indicate that the observed effects are a mosaic of phenotypes and that complete replacement of endogenous ClpX with ClpX_R230A_ would likely exacerbate the observed results. To date, we have been unable to generate a knockdown strain in which we complement back with ClpP2 and ClpX_R230A_. This dual complementation is critical, as knockdown of ClpP2 with no complementation would drastically affect the readout of any assay we perform (34). Solutions to these issues are currently under development.

While the transcript levels of *tmRNA* are high throughout most of the developmental cycle, that transcription maximally peaks at 16 hpi supports that *trans*-translation occurs extensively during peak RB growth and replication when translation is maximal. Clearly, *trans*-translation is critical to the overall physiology of C. trachomatis, as evidenced by treatment with the MBX-4132 inhibitor and by knockdown of *tmRNA* (Fig. 3 and 4). This importance is further underscored by *tmRNA* transcript levels near that of most tRNAs. This mid-developmental cycle peak in transcription supports our model for an accumulation of SsrA-tagged substrates prior to initiation and/or progression of secondary differentiation. This information, in turn, supports a model in which SsrA-tagged substrates compete with untagged targets of ClpX that ultimately leads to an accumulation of an untagged factor that triggers secondary differentiation. In further support of this model, the peak expression of ClpX is mid-developmental cycle, but ClpX expression levels remain elevated throughout the remainder of development (60). Given that ClpX appears to be intimately involved in both general chlamydial development and secondary differentiation, the correlating peak in *trans*-translation fits a model where ClpX and *trans*-translation are functionally linked. While we cannot rule out the presence of ClpX adaptors resulting in a shift of ClpX substrate preference as in *Caulobacter* species (61, 62), the apparent lack of any C. trachomatis homologs to this system suggests a more simplistic system. The identification of any noncanonical ClpX adaptor proteins throughout chlamydial development is underway in our lab. Although other proteases, including Lon (63), FtsH (64), and Tsp (8), contribute to degradation of SsrA-tagged substrates, the congruency of phenotypes between ClpX_R230A_ overexpression and *tmRNA*^6xHis^ complementation in parallel with the *in vitro* GFP_SsrA degradation assays supports that ClpXP serves as the primary factor for SsrA tag recognition and subsequent degradation in C. trachomatis, as observed in other bacteria (13).

We have successfully expanded our repertoire of tools for the genetic manipulation of C. trachomatis by introducing a system for inducible knockdown with complementation of an RNA species. Our previous study relied on the production of two gene products from one transcript. Although useful, this system prevents the complementation of a functional RNA molecule, such as *tmRNA*, that requires its tertiary structure for function (65, 66). To circumvent this requirement, we leveraged a highly active promoter to drive transcription of the *tmRNA* (55, 67). While not inducible, this construct allows for relatively constitutive complementation that, when coupled with inducible knockdown, provides a robust mechanism to study functional RNAs in C. trachomatis.

In conclusion, we have provided evidence that ClpX and *trans*-translation serve critical functions in chlamydial differentiation. This work contributes a crucial mechanistic aspect to our understanding of a fundamental facet of chlamydial biology and, within the field, builds on the innovative approaches for genetic manipulation of Chlamydia. Overall, these studies provide both an important foundation for future investigations of chlamydial differentiation and contribute a novel function to the ever-growing body of *trans*-translation research.

## MATERIALS AND METHODS

### Strains and cell culture.

McCoy fibroblasts were used for the transformation, propagation, and experimentation of the indicated strains. Clonal C. trachomatis populations were achieved using serial dilution. Cells were grown and passaged in Dulbecco’s modified Eagle’s medium (DMEM; ThermoFisher) supplemented with 10% fetal bovine serum (FBS; Sigma, St. Louis, MO). Cultures were routinely verified to be free of *Mycoplasma* contamination by 4′,6-diamidino-2-phenylindole (DAPI) staining and by using the LookOut mycoplasma PCR detection kit (Sigma). For the *trans*-translation inhibitor studies, wild-type, density gradient-purified Chlamydia trachomatis 434/Bu (ATCC VR902B) EBs were used. For chlamydial transformations, Chlamydia trachomatis serovar L2 EBs naturally lacking the endogenous chlamydial plasmid (ATCC 25667R) were used, as in reference 68.

### Plasmid construction.

The primers, gBlock gene fragments, plasmids, and bacterial strains used for molecular cloning are listed in Table S1 in the supplemental material. Constructs for chlamydial transformation were cloned using high-fidelity (HiFi) ligation independent cloning (LIC) reactions from New England BioLabs (NEB). Primers were designed using the NEBuilder online primer generation tool (https://nebuilderv1.neb.com). To introduce the R230A mutation into ClpX, primers encoding the mutation were designed such that a two ClpX fragments were produced and subsequently inserted into a derivative of the pBOMB4-Tet::L2 plasmid (kind gift of Ted Hackstadt [69]). This method was performed using both wild-type and E187A ClpX sequences from verified constructs. Cloned plasmids were confirmed by diagnostic restriction enzyme digest and Sanger sequencing. CRISPRi constructs were designed using the same methods as described previously (34). Briefly, guide RNA sequences were designed as in reference 70, which were then ordered as a gBlock gene fragment (Integrated DNA Technologies [IDT], Coralville, IA). For the knockdown with complementation plasmids, the CRISPRi constructs containing the gRNA were digested with the FastDigest restriction enzyme NheI (ThermoFisher). The promoter sequence for the *incDEF* operon and the wild-type *tmRNA* promoter, gene, and terminator were amplified by PCR and ligated into the plasmid using the HiFi reaction as above. For the *tmRNA*^6xHis^ variant, a gBlock encoding the 6×His mutation as the 5′ fragment of *tmRNA* was used, where a *tmRNA* 3′ fragment was produced by PCR. All fragments were ligated as described above. All pBOMB4 and pBOMBLCRia constructs were transformed into NEB 10β Escherichia coli for propagation.

10.1128/mbio.01858-22.4 TABLE S1 list of primers, plasmids, and strains used in the study. Download Table S1, DOCX file, 0.03 MB. Copyright © 2022 Wood et al. 2022 Wood et al. https://creativecommons.org/licenses/by/4.0/ This content is distributed under the terms of the Creative Commons Attribution 4.0 International license.

All clones containing either pLATE31 (clpX) or pLATE52 (gfp) were generated using the ligation independent cloning (LIC) method as directed (Thermo Scientific). Genes were amplified by PCR using Phusion high-fidelity PCR master mix with their respective primers. Upon amplification, products were run on an agarose gel, visualized using ethidium bromide staining and UV transillumination, and then extracted and purified using the GeneJET gel extraction kit (Thermo Scientific). The PCR/pLATE product was then used for chemical transformation into the respective E. coli cloning strain. Transformants were subjected to colony PCR using Fermentas master mix (Thermo Scientific) and the respective primers to confirm insertion of the gene into the vector. PCR-confirmed clones were struck out for isolation onto 100 μg/mL ampicillin LB plates and grown in LB broth supplemented with ampicillin, and the plasmid was isolated using the GeneJET plasmid miniprep kit (Thermo Scientific). Upon sequence verification of the insert (performed by Psomagen), plasmids were transformed via electroporation into E. coli BL21(DE3) ΔPAX.

### Recombinant protein purification.

C-terminal 6×His-tagged C. trachomatis ClpX and ClpX mutants were purified from 500-mL cultures of E. coli BL21(DE3) ΔPAX containing the respective plasmids as described in Wood et al. (60). Briefly, cultures were induced for 20 h at 18°C with 0.5 mM isopropyl-β-d-thiogalactopyranoside (IPTG). After induction, cultures were pelleted and stored at −80°C overnight. Samples were suspended in ClpX lysis/wash buffer (25 mM Tris base [pH 7.5], 300 mM NaCl, and 10 mM imidazole), sonicated, spun down to separate the nonsoluble and soluble fractions, filtered using 0.45-μm filters, and rotated end over end for 1 h with HisPur cobalt resin (Thermo Scientific). After binding, the resin was washed using the ClpX lysis/wash buffer to remove contaminating proteins. Proteins were then eluted from the resin using the ClpX elution buffer (25 mM Tris base [pH 7.5], 300 mM NaCl, and 300 mM imidazole) and were collected. After four elution steps, the buffer was exchanged to ATPase assay buffer (25 mM HEPES [pH 7.2], 200 mM KCl, 20 mM MgCl_2_, and 10% glycerol) using a Millipore Amicon Ultra 15 filtration units (3-kDa cutoff). ClpX proteins were then quantified using the Bio-Rad protein assay, assessed for purity on 10% SDS-PAGE gels with Coomassie brilliant blue staining, and identified using anti-His-tag Western blot. Blotting was performed using a mouse monoclonal anti-6×His antibody (1:1,000; Millipore; HIS.H8) and a goat anti-mouse IgG horseradish peroxidase (HRP)-conjugated secondary antibody (1:2,000; MilliporeSigma; AP124P). Protein samples were aliquoted and stored at −80°C.

N-terminal 6×His-tagged GFP with a C-terminal SsrA tag was purified from a 500-mL culture of E. coli BL21(DE3) ΔPAX. Cultures were induced for 6 h at room temperature with 0.5 mM IPTG. After induction, cultures were pelleted and stored at −80°C overnight. Samples were resuspended in GFP lysis/wash buffer (50 mM NaH_2_PO_4_ [pH 8], 300 mM NaCl, and 20 mM imidazole), sonicated, spun down to separate the nonsoluble and soluble fractions, filtered using 0.45-μm filters, and rotated end over end for 1 h with HisPur cobalt resin (Thermo Scientific). After binding, the resin was washed using the GFP lysis/wash buffer to remove contaminating proteins. Proteins were then eluted from the resin using the GFP elution buffer (50 mM NaH_2_PO_4_ [pH 8], 300 mM NaCl, and 300 mM imidazole) and collected. After four elution steps, the buffer was exchanged to GFP storage buffer (50 mM NaH_2_PO_4_ [pH 8], 300 mM NaCl, and 10% glycerol) using a Millipore Amicon Ultra 15 filtration units (3-kDa cutoff). GFP proteins were then quantified using the Bio-Rad protein assay, assessed for purity on 12% SDS-PAGE gels with Coomassie brilliant blue staining, and identified using anti-His-tag Western blot. Blotting was performed using a mouse monoclonal anti-6×His antibody (1:1,000; Millipore; HIS.H8) and a goat anti-mouse IgG HRP-conjugated secondary antibody (1:2,000; MilliporeSigma; AP124P). Fluorescence was confirmed by spotting samples onto slides for fluorescence microscopy and in 96-well plates for analysis with a BioTek Synergy H1 plate reader at excitation λ of 465 nm and emission λ of 535 nm. Protein samples were aliquoted and stored at −80°C.

### Kinase-GLO ClpX ATPase assay.

Recombinant ClpX or mutant ClpX (1.5 μg) was incubated in up to 49.5 μL of ATPase assay buffer without ATP for 10 min at room temperature. To initiate the reaction, 1 μM ATP dissolved in ATPase assay buffer was added to the reaction to give a final volume of 50 μL and incubated at 30°C for 1.5 h. After the 1.5-h incubation, the reaction mixtures were incubated for an additional 30 min at room temperature. Fifty microliters of the Kinase-Glo reagent (Promega) was added to the reaction mixture, and it was incubated for 10 min at room temperature. Using a BioTek Synergy H1 plate reader, the luminescence of the reaction was measured (ATP not consumed by ClpX). The assays were performed three independent times for three biological replicates.

### GFP degradation assay.

ClpP1 and ClpP2 at 3.5 μM (purified as performed in Wood et al. [60] using the E. coli BL21(DE3) ΔPAX strain) and 3 μM ClpX were incubated with 4 mM ATP, 8 mM creatine-phosphate, 10× assay buffer (1× final; 25 mM HEPES [pH 7.6], 200 mM KCl, 5 mM MgCl_2_, 1 mM dithiothreitol [DTT], and 10% glycerol), and 1 U/mL creatine phosphokinase (MilliporeSigma) for 30 min at 32°C. GFP at 0.36 μM was then added to the reaction for a final volume of 100 μL, samples were mixed via pipetting, and fluorescence was read using a prewarmed (32°C) BioTek Synergy H1 plate reader for 2 h at excitation λ of 465 nm and emission λ of 535 nm using a flat-bottom white plate. Assays were performed three times for three biological replicates.

### ClpX oligomerization assay.

Five micrograms of purified ClpX wild-type or mutant proteins was incubated with oligomerization buffer (25 mM Tris base [pH 7.5], 5 mM KCl, 5 mM MgCl_2_, 1 mM DTT, and 10% glycerol) at 37°C for 20 min. Reaction mixtures were then combined with 5× native sample buffer without heating and run on Bio-Rad MiniProtean 4% to 20% gradient gels for native-PAGE. NativeMark (Invitrogen) unstained protein standards were used as molecular weight markers. Protein was detected using Coomassie brilliant blue staining, and gels were imaged using a Bio-Rad Chemidoc MP. The image shown in Figure 1A is representative of three biological replicates.

### Chlamydial transformation.

Chlamydial transformation was performed using an adaptation of previously described protocols (68, 71). Briefly, ~2 μg of sequence-verified constructs was incubated with 10^6^ C. trachomatis serovar L2 (CtrL2) EBs lacking the endogenous plasmid (ATCC 25667R) in 50 μL of CaCl_2_ at room temperature for 30 min. One well of a 6-well plate with a fresh monolayer of McCoy mouse fibroblasts was infected with one of the indicated reaction mixtures. Samples were infected by centrifugation, and antibiotic-free DMEM was replaced at 8 hpi with DMEM containing 1 μg/mL of cycloheximide and 1 U/mL of penicillin G. Infected cells were then passaged every 48 h until a population of GFP-positive, penicillin-resistant C. trachomatis was established. Samples were then serially diluted to isolate clonal populations. These isolated populations were then expanded and frozen at −80°C in a sucrose phosphate solution (2SP). To confirm plasmid sequences, DNA was harvested from infected cultures and was transformed into NEB 10β E. coli for plasmid propagation. Plasmids isolated from these transformants were then verified by restriction digest and sequencing.

### Determination of developmental effects by immunofluorescence assay (IFA) and inclusion forming units (IFUs).

To determine the effects of overexpression or knockdown on C. trachomatis, both indirect immunofluorescence assays (IFA) and inclusion forming unit (IFU) assays were utilized. For the IFA assays, the ClpX_R230A_ overexpression and the *tmRNA* knockdown-only samples were fixed using formaldehyde-glutaraldehyde in Dulbecco's phosphate-buffered saline (DPBS) for 5 min to maintain a GFP signal. Samples were then permeabilized with 100% MeOH and probed using a goat anti-major outer membrane protein (MOMP; Meridian, Cincinnati, OH) primary antibody and either a primary rabbit anti-6×His (Abcam, Cambridge, MA) for ClpX samples or primary rabbit anti-dCas9 (Abcam) for knockdown samples. Samples were then stained with the appropriate secondary antibody (Invitrogen, Carlsbad, CA) and DAPI for visualization. Coverslips were then mounted on glass slides using ProLong glass antifade mounting media (Invitrogen) and imaged on a Zeiss Axio imager Z.2 equipped with Apotome.2 optical sectioning hardware and X-Cite Series 120PC illumination lamp. For IFU analysis, samples were infected and then induced and harvested as indicated. At the indicated time point, three wells of a 24-well plate were scraped in 2SP, vortexed with 1 mM glass beads, and frozen at −80°C. Samples were then serially diluted onto a fresh monolayer of cells, allowed to grow for 24 h, fixed, and enumerated. All experiments were performed three times for three biological replicates.

### Genomic DNA level analysis.

To assess genomic DNA (gDNA) content following overexpression or knockdown, one well of a 6-well plate per condition was infected with the indicated strain, harvested in DPBS at the given time point, and frozen at −80°C. Each sample was then thawed and frozen twice more for a total of three freeze/thaw cycles, after which gDNA was extracted using the DNeasy DNA extraction kit (Qiagen). The resulting gDNA samples were then diluted down to 5 ng/μL prior to use in quantitative PCR (qPCR). Five microliters of the diluted samples (25 ng of total gDNA) was then mixed with 20 μL of a PowerUp Sybr green PCR master mix in a 96-well qPCR plate and was analyzed on a QuantStudio 3 system (Applied Biosystems). Each sample from each biological replicate was tested in triplicate. A standard curve of CtrL2 genomic DNA was used for interpolation of acquired cycle threshold (*C_T_*) values. All experiments were performed three times for three biological replicates.

### Analysis of HctB levels.

An assessment of HctB levels following the overexpression or knockdown was performed as per the protocol described in reference 34. Briefly, a six-well plate was infected with the indicated strains and was induced or not. Each well was lysed using a urea-based buffer (8M urea, 10 mM Tris, 2.5% β-mercaptoethanol, and 0.1% SDS with Pierce universal nuclease added immediately prior to use). Protein concentrations were determined using the Pierce EZQ protein quantitation kit per the manufacturer’s instructions, after which 50 μg of protein was separated by SDS-PAGE and transferred to a polyvinylidene difluoride (PVDF) membrane. Blots were then probed with both goat anti-MOMP and rabbit anti-HctB (generous gift from Ted Hackstadt, NIH) primary antibodies, rinsed, and stained with both donkey anti-goat 680 (Li-Cor, Lincoln, NE) and donkey anti-rabbit 800 (Li-Cor) secondary antibodies. Blots were imaged on an Azure c600 imaging system with care taken to prevent saturation of the band intensities. The integrated density of each band was measured using FIJI software (72), after which the ratios of HctB to MOMP were determined. The HctB/MOMP ratios of the induced and uninduced samples were then compared for normalization to the uninduced samples prior to analysis. The experiment was performed three times for three biological replicates, and the image shown is representative of three biological replicates.

### RNA extraction and RT-qPCR to assess transcripts following ClpX_R230A_ overexpression.

For each condition, RNA was extracted from one well of a six-well plate using TRIzol (Invitrogen) and chloroform per the manufacturer’s recommendation. In parallel, another well was harvested and processed for gDNA using the DNeasy kit (Qiagen). A total of 10 μg of RNA was DNase-treated with the Turbo DNA-free kit (Ambion), precipitated, rinsed, and resuspended. One microgram of the resulting DNase-treated RNA was used to generate cDNA using SuperScript III reverse transcriptase (Invitrogen). The resulting cDNA was diluted and frozen at −80°C prior to use. A total of 5 μL of each sample was used per well of a 96-well qPCR plate. For each of three biological replicates, each sample was analyzed in triplicate using the QuantStudio 3 system. Levels of gDNA were determined as described above. For each primer set used, a standard curve of gDNA was generated for quantitation, and the RNA levels were normalized to gDNA levels for analysis. All experiments were performed three times for three biological replicates.

### Confirmation of *tmRNA* knockdown and complementation.

Knockdown and subsequent complementation of *tmRNA* was confirmed by RT-qPCR following induction or not. Samples were infected and then induced or not. RNA and gDNA were extracted, processed, and analyzed as describe above, where transcript abundances were compared with the levels of gDNA. Additionally, strains were monitored for morphological differences by IFA. Samples were fixed with MeOH to eliminate GFP fluorescence and stained with goat anti-MOMP (Meridian), rabbit anti-Sa_dCAS9 (Abcam), and mouse anti-6×His (GenScript) primary antibodies. Samples were then stained with the appropriate secondary antibodies (Invitrogen) and DAPI (Sigma), mounted using ProLong glass antifade mounting media (Invitrogen), and imaged as described previously. The image shown is representative of three biological replicates.

### Determination of the effect of *trans*-translation inhibition on chlamydial development.

Stocks of the *trans*-translation inhibitor MBX-4132 (MedChemExpress; Monmouth Junction, NJ; catalog [cat] number HY-112565) were resuspended in dimethyl sulfoxide (DMSO) at 10 mM and frozen at −80°C. An inhibitory concentration of MBX-4132 on C. trachomatis was determined to be 50 μM by dose curve. Limited inhibition of C. trachomatis was observed at 25 μM or lower concentrations of MBX-4132. For the 24-h samples, 0.5 mL of DMEM containing 50, 25, or 12.5 μM MBX-4132 was added at 8 hpi, and samples were harvested or fixed at 24 hpi. MBX-4132 concentrations of 50 μM or 25 μM in 500 μL of DMEM was added to the 48-h samples at 8 hpi, which were harvested or fixed at 48 hpi. For the reactivation samples, MBX-4132 at 50 μM or 25 μM was added at 8 hpi. At 24 hpi, the compound was removed and fresh DMEM was added after a rinse step with Hank’s balanced salt solution (HBSS). After an additional 24 h, the reactivation samples were harvested and fixed. To observe the impact of MBX-4132 on preformed EBs, compound concentrations of 50 μM or 25 μM were added at 24 hpi, and samples were harvested or fixed at 48 hpi. To harvest EBs of like samples, three wells of a 24-well plate were scraped and pooled in 2SP, vortexed with three 1-mm glass beads, and frozen at −80°C. These samples were titrated for secondary infection onto a fresh monolayer of HEp2 cells, fixed with no additional treatment at 24 h, and analyzed according to the IFU assay. Methanol fixation was used to fix morphology samples, which were imaged as described above after antibody staining. Each experiment was performed three times for three biological replicates.
