# Supplementary figures and images for: Tag-Dependent Substrate Selection of ClpX Underlies Secondary Differentiation of Chlamydia trachomatis
Source: mBio. 2022 Sep 26;13(5):e01858-22. doi: 10.1128/mbio.01858-22 (PMC9601184; doi:10.1128/mbio.01858-22)

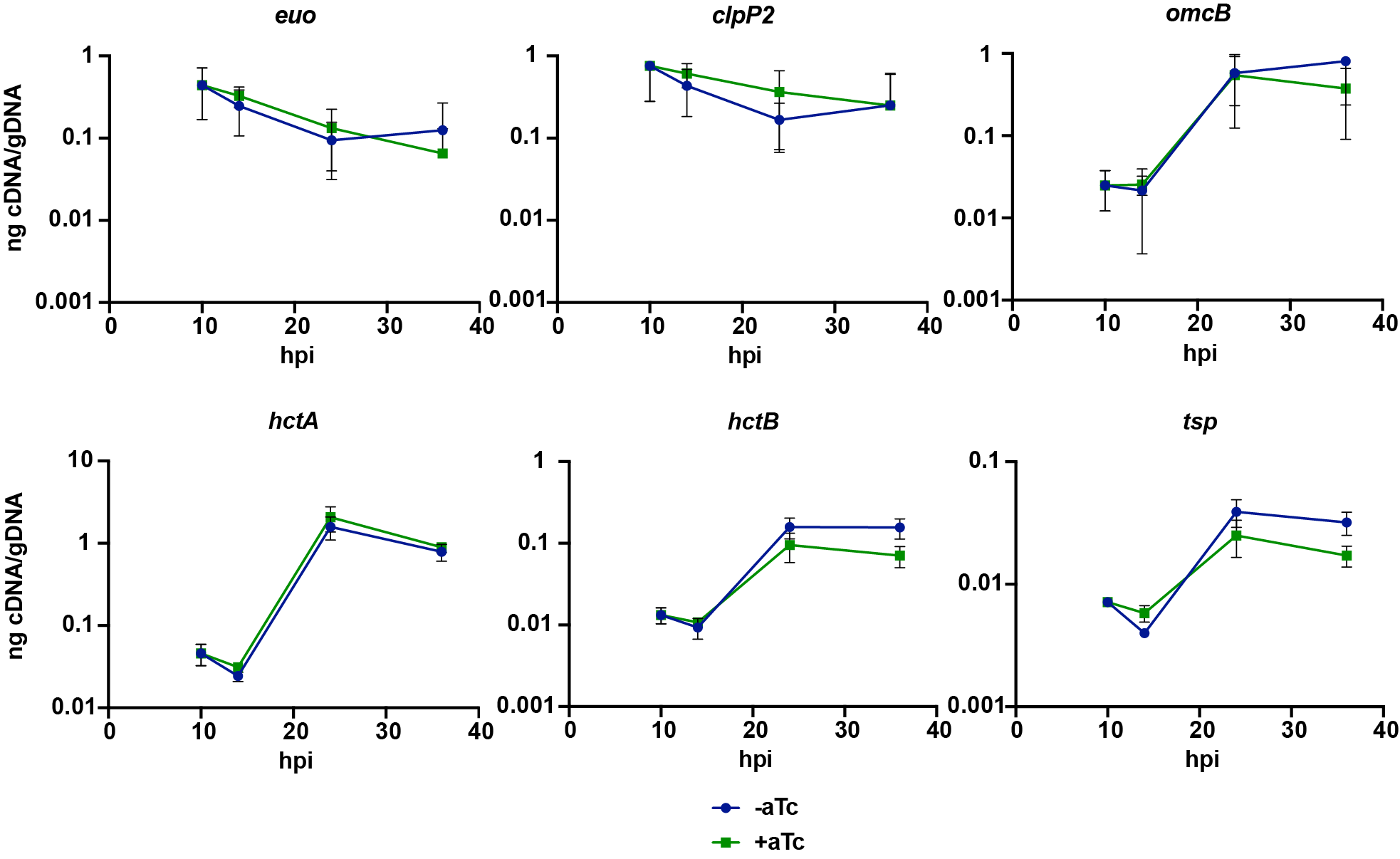

Supplement: FIG S1 [file mbio.01858-22-s0001.tif]

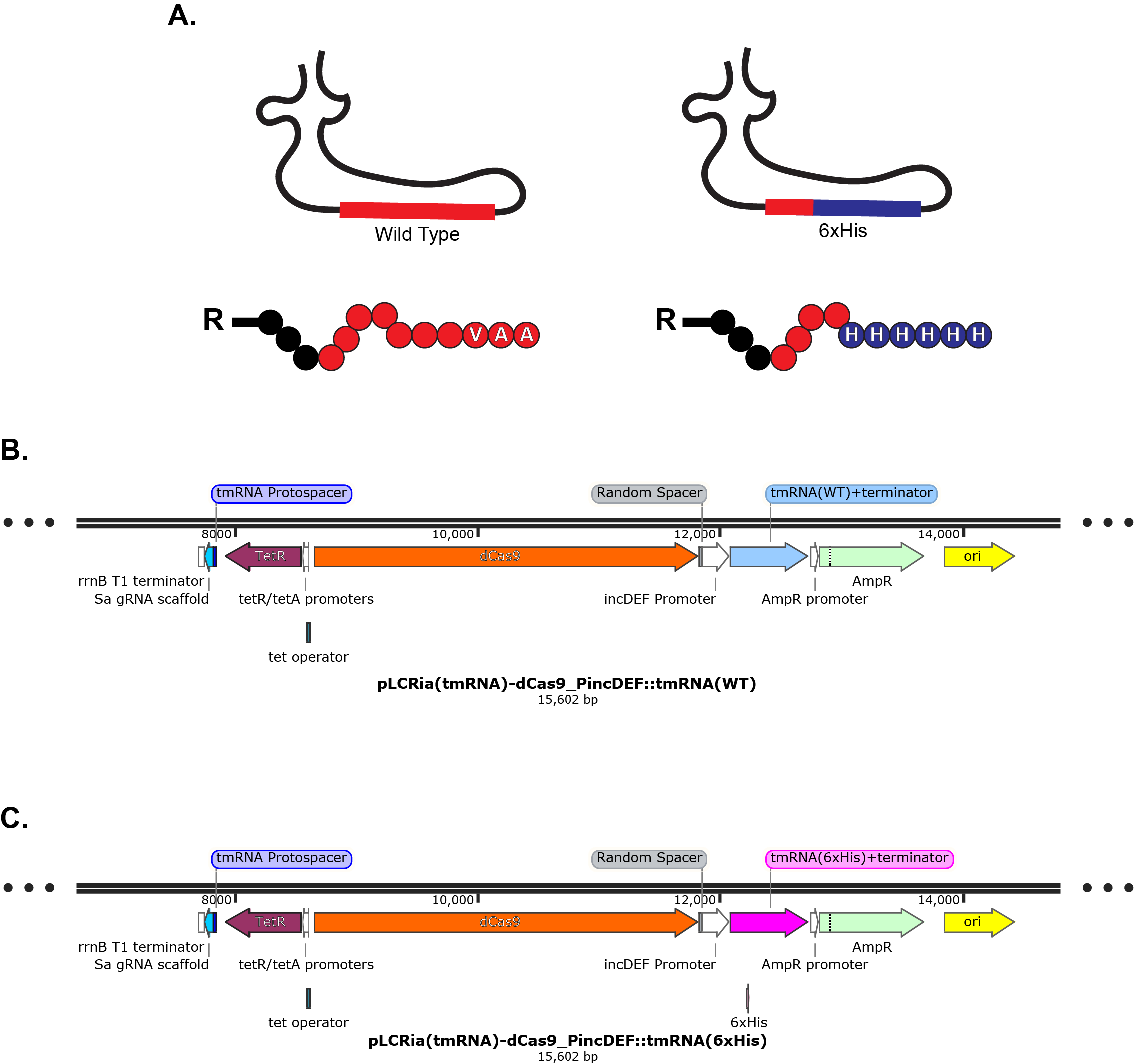

Supplement: FIG S2 [file mbio.01858-22-s0002.tif]

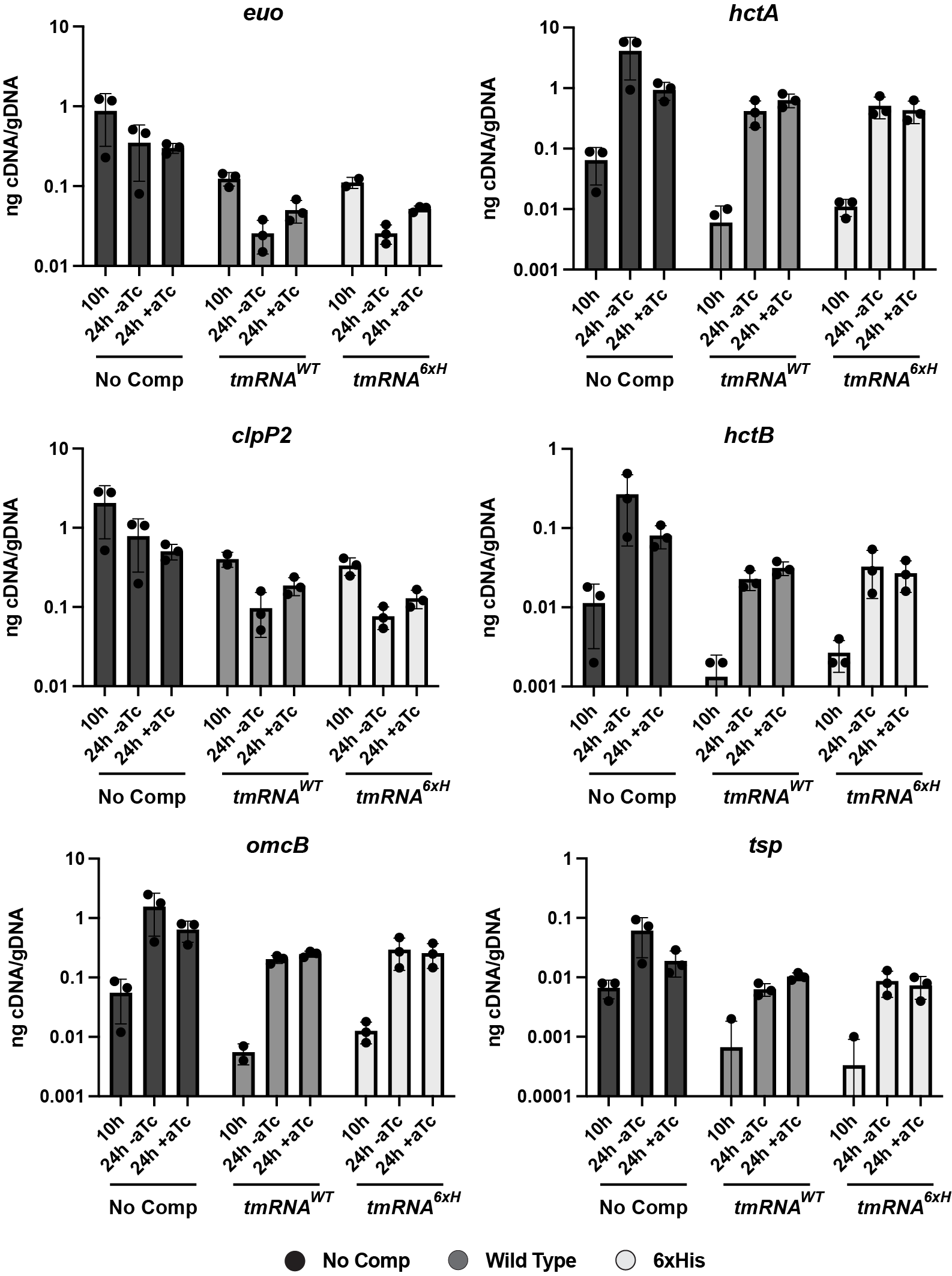

Supplement: FIG S3 [file mbio.01858-22-s0003.tif]
