# Supplementary material for: Tag-Dependent Substrate Selection of ClpX Underlies Secondary Differentiation of Chlamydia trachomatis
Source: mBio. 2022 Sep 26;13(5):e01858-22. doi: 10.1128/mbio.01858-22 (PMC9601184; doi:10.1128/mbio.01858-22)
Supplement: TABLE S1 [file mbio.01858-22-s0004.docx]

**Supplementary table 1. The list of Plasmids, Strains, and Primers**

| **Construct Plasmid** | **Relevant genotype** | **Ori** | **Source of Reference** |
| --- | --- | --- | --- |
| pBOMB4-*clpX_R230A_* | *bla* P*tet*::*clpX_R230A__*6xH | pUC19 | This study |
| pBOMB4-*clpX_R230A/E187A_* | *bla* P*tet*::*clpX_R230A/E187A__*6xH | pUC19 | This study |
| pLATE31-*clpX*_R230A__6xH | *bla* P*_tet_* P*_lac_*::*clpX_R230A_*_6xH | pMB1 | This study |
| pLATE31-*clpX*_R230A/E187A__6xH | *bla* P*_tet_* P*_lac_*::*clpX_R230A/E187A_*_6xH | pMB1 | This study |
| pLATE31-*clpX_E187A_*_6xH | *bla* P*_tet_* P*_lac_*::*clpX_E187A_*_6xH | pMB1 | (1) |
| pLATE52-6xH_*gfp(ssrA)* VAA | *bla* P*_tet_* P*_lac_*::6xH_*gfp(ssrA)* VAA | pMB1 | This study |
| pLATE52-6xH_*gfp*(*ssrA*) VDD | *bla* P*_tet_* P*_lac_*::6xH_*gfp(ssrA)* VDD | pMB1 | This study |
| pLATE31-*clpP1*_6xH | *bla* P*_tet_* P*_lac_*::*clpP1*_6xH | pMB1 | (1) |
| pLATE31-*clpP2*_6xH | *bla* P*_tet_* P*_lac_*::*clpP2*_6xH | pMB1 | (1) |
| pLATE31-*clpX*_6xH | *bla* P*_tet_* P*_lac_*::*clpX*_6xH | pMB1 | (1) |
| pLCRia(*tmRNA*)::L2 | *bla* P_dnaKm_::*tmRNA*_gRNA P*_tet_*::*Sa_dCas9vaa* | pUC19 | This study |
| pLCRia(*tmRNA*)-*tmRNA*^WT^::L2 | *bla* P_dnaKm_::*tmRNA*_gRNA P*_tet_*::*Sa_dCas9vaa* P_incDEF_::*tmRNA*^WT^ | pUC19 | This study |
| pLCRia(*tmRNA*)-*tmRNA*^6xHis^::L2 | *bla* P_dnaKm_::*tmRNA*_gRNA P*_tet_*::*Sa_dCas9vaa* P_incDEF_::*tmRNA*^6xHis^ | pUC19 | This study |

| ***E. coli* Strain** | **Relevant genotype** | **Source of Reference** |
| --- | --- | --- |
| DH5⍺ | *fhuA2 Δ(argF-lacZ)U169 phoA glnV44 Φ80 Δ(lacZ)M15 gyrA96 recA1 relA1 endA1 thi-1 hsdR17* | New England BioLabs |
| DH10β | *Δ(ara-leu) 7697 araD139 fhuA ΔlacX74 galK16 galE15 ϕ80dlacZΔM15 (e14-) recA1 relA1 endA1 nupG rpsL (*Str^R^*) rph spoT1 Δ(mrr-hsdRMS-mcrBC)* | New England BioLabs |
| *E*. *coli* dAPX-1 | *fhuA2, [lon], ompT, gal, [dcm], ΔhsdS, λ DE3 (λ sBamHIo ΔEcoRI-B int::(lacI::PlacUV5::T7 gene1) i21 Δnin5)* ΔclpX clpP::cam clpA::kan | (2) |

| **Primer name** | **Sequence** | **Features** | **Usage** |
| --- | --- | --- | --- |
| Ct443 *omcB* | CGGTAGGATCTCCCTATCCTATT | Forward qPCR primer | For qPCR of *omcB* |
| Ct443 *omcB* | CGAACTCTGCTTCACATGGTA | Reverse qPCR primer | For qPCR of *omcB* |
| Ct446 *euo* | CGAAGACTACTCGTTGGGAAATA | Forward qPCR primer | For qPCR of *euo* |
| Ct446 *euo* | AACAGAAGCTCTCCTTGATAAGT | Reverse qPCR primer | For qPCR of *euo* |
| Ct706 *clpP_2* | GTTAGCGATTTACGACACCATTC | Forward qPCR primer | For qPCR of *clpP2* |
| Ct706 *clpP_2* | CCCTTTGTCCCTGCAGATAATA | Reverse qPCR primer | For qPCR of *clpP2* |
| Ct743 *hctA* | AAGCTAAAGCTGCTGCTAAGA | Forward qPCR primer | For qPCR of *hctA* |
| Ct743 *hctA* | GTTGGTTTGACCTTTGCTTTAGT | Reverse qPCR primer | For qPCR of *hctA* |
| Ct046 *hctB* | AACTGTAGCAGCTCGTAAGC | Forward qPCR primer | For qPCR of *hctB* |
| Ct046 *hctB* | TTTGCGAGCTACAGTCTTCTT | Reverse qPCR primer | For qPCR of *hctB* |
| Ct441 *tsp* | GTAGCCTTCGTGTAGGTGATATT | Forward qPCR primer | For qPCR of *tsp* |
| Ct441 *tsp* | AGGATCCTGGAGAACCTCTT | Reverse qPCR primer | For qPCR of *tsp* |
| Ct_s01 *tmRNA* | GGGTGTAAAGGTTTCGACTTAGA | Forward qPCR primer | For qPCR of *tmRNA* |
| Ct_s01 *tmRNA* | AGCCTTAGGTTCGGCATTTAT | Reverse qPCR primer | For qPCR of *tmRNA* |
| pBOMB4/*clpX*/LIC Forward | ttaactttaagaaggagataATGACAAAAAAAAATCTTGCGGTC | Lowercase for plasmid overlap construction | For amplification of *clpX* for insertion into pBOMB4 |
| *clpX*_5’ Frag/LIC Reverse | tagctcctcctttAGGAGGGATATTTGCAACAGTTC | Lowercase for 3’ fragment overlap | For amplification of *clpX* 5’ fragment with R230A mutation |
| *clpX*_3’ Frag/LIC Forward | tatccctcctAAAGGAGGAGCTAAGCATC | Lowercase for 5’ fragment overlap | For amplification of *clpX* 3’ fragment with R230A mutation |
| *clpX*/6xHis/pBOMB4/LIC Reverse | tttcacttcacaggtcaaccttagtgatggtgatggtgatgAGCAATCGCCTCTGGTGATTTC | Lowercase for plasmid overlap, underline for 6xHis sequence | For amplification of *clpX* with fused 6xHis tag for pBOMB4 insertion |
| 0074F/LIC Forward | agaaggagatataactatgACAAAAAAAAATCTTGCGGTCTGTTC | Lowercase for plasmid overlap construction | For amplification of mutant *clpX* templates from pBOMB4 vectors ; inserted into pLATE31 |
| 0074R/LIC Reverse | gtggtggtgatggtgatggcCAGCAATCGCCTCTGGTGATTTC | Lowercase for plasmid overlap construction | For amplification of mutant *clpX* templates from pBOMB4 vectors ; inserted into pLATE31 |
| pLATE52 *gfp* F | ggttgggaattgcaaAGTAAAGGAGAAGCACTTTTCACTGGAG | Lowercase for plasmid overlap construction | For amplification of *gfp*(*ssrA*); inserted into pLATE52 |
| pLATE52 *gfp* VAA | ggagatgggaagtcattaTTAAGCAGCTACGCGTAGATC | Lowercase for plasmid overlap construction | For amplification of *gfp*(*ssrA*) VAA; inserted into pLATE52 |
| pLATE52 *gfp* VDD | ggagatgggaagtcattaTTAATCATCTACGCGTAGATCTTCGAGATC | Lowercase for plasmid overlap construction | For amplification of *gfp*(*ssrA*) VDD; inserted into pLATE52 |
| incDEF_Prom/*tmRNA*/pLCRia Forward | aagtgaggttGGGGGTGTAAAGGTTTCG | Lowercase for gBlock overlap | For amplification of *tmRNA* for pLCRia(*tmRNA*)-*tmRNA*::L2 construction |
| *tmRNA*/pLCRia(Nhe)/LIC Reverse | ttgaatggtcgaccggtacgCTTAAAAAGGTAAAACTTCTAGCTTTTG | Lowercase for plasmid overlap | For amplification of *tmRNA* for pLCRia(*tmRNA*)-*tmRNA*::L2 construction |
| *tmRNA*_180_5' Frag Reverse | tcacccgtatTTAGGTAACAACTTTGCTAATTAGTG | Lowercase for 3’ fragment overlap | For amplification of *tmRNA*^6xHis^ from gBlock |
| *tmRNA*_3' Frag Forward | tgttacctaaATACGGGTGACCCGGTGTTC | Lowercase for 5’ fragment overlap | For amplification of *tmRNA* from gDNA |

| **gBlock Name** | **Sequence** | **Features** | **Usage** |
| --- | --- | --- | --- |
| *tmRNA*_IGR gRNA gBlock | *tgtgaaagtgggtcttaagacgtcg*gtactgcatgtgacgcacgtagatcatgcaTTCACCGGTGGAGACGGTTTTCTTATAATGACACC**AATCCTTACAATCCTCAGCTC**GTTTTAGTACTCTGGAAACAGAATCTACTAAAACAAGGCAAAATGCCGTGTTTATCTCGTCAACTTGTTGGCGAGATTTTTCAAATAAAACGAAAGGCTCAGTCGAAAGACTGGGCCTTTCGTTTTATcaacagcggtctactgaatctgagctagtg*cgtgatataattaaaattatattca* | Italicized sequence represents pBOMB flanking regions for HiFi insertion.  Lowercase sequence is random spacer.  Bolded sequence is the target gRNA. | For CRISPRi knockdown of *tmRNA* |
| *tmRNA* (1-180)+6xHis gBlock | gttctaaccagatacatgacggtgtttcgaaaacctctggtggagctcgcgaacaccgggtcacccgtatttaggtaacaactttgctaatta**GTGATGGTGATGGTGATG**ttcgagatcagcgaagctgataatttcgcattcagccttaggttcggcatttattgttttgtcggctttttaggaggccagccaacgccctccgcatgcaattaacgcttcatttctaagtcgaaacctttacacccccgtttaagctc | Bolded uppercase represents 6xHis replacement of SsrA tag | Template for addition of 6xHis mutation into *tmRNA* |
| P_incDEF_ +Random spacer for pLCRia (NheI) *tmRNA* Complementation | aaagggcgtagcagcataagAATAGGGATAAACATACAAGTCGATAGAAAAACGGAGCCTTCTAGCTATTTTGTAAATATTTTAACAATTTAGATTCTTCAAAGCTCAGCGAGGGCGTGAAGAATCTTGTTCAGGTGTATTTGAAAAAAGTTTGTTTTAAATAGTTTTTTTAGTTAAAATGGGTCCCTAAATAATTTAAATCCGGTAGTTTTTGCGTCCGAAACATTGTTTTATAAGTGAGAAATGAGATCTGGCTAAAATCTGTCGAAGTGAGGTTgggggtgtaaaggtttcg | Lowercase represents overlaps for LIC. Random spacer underlined. | gBlock for *incDEF* promoter to drive *tmRNA* transcription during complementation |
| *gfp*(*ssrA*) VAA | GAAGTTCTGTTCCAGGGGCCCCTGGGATCCAGTAAAGGAGAAGCACTTTTCACTGGAGTTGTCCCAATTCTTGTTGAATTAGATGGTGATGTTAATGGGCACAAATTTTCTGTCAGTGGAGAGGGTGAAGGTGATGCAACATACGGAAAACTTACCCTTAAATTTATTTGCACTACTGGAAAACTACCTGTTCCATGGCCAACACTTGTCACTACTCTTACGTATGGTGTTCAATGCTTTTCAAGATACCCAGATCATATGAAACGGCATGACTTTTTCAAGAGTGCCATGCCCGAAGGTTATGTACAGGAAAGAACTATATTTTTCAAAGATGACGGGAACTACAAGACACGTGCTGAAGTCAAGTTTGAAGGTGATACCCTTGTTAATAGAATCGAGTTAAAAGGTATTGATTTTAAAGAAGATGGAAACATTCTTGGACACAAATTGGAATACAACTATAACTCACACAATGTATACATCATGGCAGACAAACAAAAGAATGGAATCAAAGTTAACTTCAAAATTAGACACAACATTGAAGATGGAAGCGTTCAACTAGCAGACCATTATCAACAAAATACTCCAATTGGCGATGGCCCTGTCCTTTTACCAGACAACCATTACCTGTCCACACAATCTGCCCTTTCGAAAGATCCCAACGAAAAGAGAGACCACATGGTCCTTCTTGAGTTTGTAACAGCTGCTGGGATTACACATGGCATGGATGAACTATACAAGGCCGAACCGAAGGCTGAATGCGAAATTATCAGCTTCGCTGATCTCGAAGATCTACGCGTAGCTGCTTAAGTCGACTCGAGCGGCCGCATCGTGACTGAC | *ssrA* VAA tag underlined | Template for cloning *gfp*(*ssrA*) VAA into pLATE52 |
| *gfp*(*ssrA*) VDD | TCCAGGGGCCCCTGGGATCCAGTAAAGGAGAAGCACTTTTCACTGGAGTTGTCCCAATTCTTGTTGAATTAGATGGTGATGTTAATGGGCACAAATTTTCTGTCAGTGGAGAGGGTGAAGGTGATGCAACATACGGAAAACTTACCCTTAAATTTATTTGCACTACTGGAAAACTACCTGTTCCATGGCCAACACTTGTCACTACTCTTACGTATGGTGTTCAATGCTTTTCAAGATACCCAGATCATATGAAACGGCATGACTTTTTCAAGAGTGCCATGCCCGAAGGTTATGTACAGGAAAGAACTATATTTTTCAAAGATGACGGGAACTACAAGACACGTGCTGAAGTCAAGTTTGAAGGTGATACCCTTGTTAATAGAATCGAGTTAAAAGGTATTGATTTTAAAGAAGATGGAAACATTCTTGGACACAAATTGGAATACAACTATAACTCACACAATGTATACATCATGGCAGACAAACAAAAGAATGGAATCAAAGTTAACTTCAAAATTAGACACAACATTGAAGATGGAAGCGTTCAACTAGCAGACCATTATCAACAAAATACTCCAATTGGCGATGGCCCTGTCCTTTTACCAGACAACCATTACCTGTCCACACAATCTGCCCTTTCGAAAGATCCCAACGAAAAGAGAGACCACATGGTCCTTCTTGAGTTTGTAACAGCTGCTGGGATTACACATGGCATGGATGAACTATACAAGGCCGAACCGAAGGCTGAATGCGAAATTATCAGCTTCGCTGATCTCGAAGATCTACGCGTAGATGATTAAGTCGACTCGAGCGGCCGCATCGTGACTGAC | *ssrA* VDD tag underlined | Template for cloning *gfp*(*ssrA*) VDD into pLATE52 |

1. Wood NA, Chung K, Blocker A, Rodrigues de Almeida N, Conda-Sheridan M, Fisher DJ, Ouellette SP. 2018. Initial Characterization of the Two ClpP Paralogs of *Chlamydia trachomatis* Suggests Unique Functionality for Each. *Journal of Bacteriology*.
2. Pan S, Malik IT, Thomy D, Henrichfreise B, Sass P. 2019. The functional ClpXP protease of *Chlamydia trachomatis* requires distinct clpP genes from separate genetic loci. *Scientific Reports* 9:14129.
